# Processing Enhances Coix Seed Prolamins Structure and Releases Functional Peptides after Digestion: In Silico and In Vitro Studies

**DOI:** 10.3390/foods12132500

**Published:** 2023-06-27

**Authors:** Shu Zhang, Zhi-Ming Li, Yu-Chao Feng, Chang-Yuan Wang, Dong-Jie Zhang

**Affiliations:** 1 College of Food, Heilongjiang Bayi Agricultural University, Xinfeng Lu 5, Daqing 163319, China; zshu996@163.com (S.Z.); lizhiming1998@126.com (Z.-M.L.); byndwcy@163.com (C.-Y.W.); 2 National Coarse Cereals Engineering Research Center, Daqing 163319, China; 3 Key Laboratory of Agro-Products Processing and Quality Safety of Heilongjiang Province, Daqing 163319, China

**Keywords:** coix seed prolamins, thermal processing, DPP-IV inhibitory activity, in silico model, simulated digestion

## Abstract

Dipeptidyl peptidase-IV (DPP-IV) is a key target for the treatment of type 2 diabetes mellitus. It is possible that peptides that precisely regulate DPP-IV could be released from coix seed prolamins (CSP), but whether this happens has not yet been investigated. We performed the in silico digestion of CSP and predicted the bioactivity, absorption, transport, toxicity, and allergenicity of the resulting peptides. The simulation predicted that 47 non-toxic bioactive peptides would be released. After screening these, we found that 64.58% of them could possess DPP-IV inhibitory activity. The effect of thermal processing on the amino acid composition and structural properties of CSP was determined, and the DPP-IV inhibitory activity of its digestion-derived peptides was also assessed. The results showed that processing could change the flavour of coix seed and the supply of amino acids. After processing, the spatial conformation of CSP changed from ordered to disordered, and the peptide content and the DPP-IV inhibitory activity of its digestion products significantly increased by 19.89–30.91% and 36.84–42.02%, respectively. These results support the hypothesis that processing can change the protein structure and increase the probability that bioactive peptides will be released. They also have important implications for the development of bioactive peptides and the intensive processing of coix seeds.

## 1. Introduction

Coix seed (*Coix lacroyma-jobi* L.) is used both as a coarse cereal in food and as a drug. It tonifies the spleen and inhibits dampness, and is also known as “The Gramineae of Life and Health” [1]. Coix seed is rich in proteins (albumins, globulins, prolamins, and glutelins [2]), active peptides (antihypertensive peptides [3], liver protective peptide [4], etc.), and essential amino acids (leucine, phenylalanine, threonine, etc. [5]) that the body can easily absorb and use. These components make it nutritious and underpin its pharmacological effects [5]. The main storage protein in coix seed is prolamin, a major high-quality plant protein that accounts for 68% of its total protein content.

Two thermal processing methods, stir-frying (SF) and stir-frying in rice bran (BSF), are common pretreatments for coix seed to be used as food [6]. Several studies have shown that thermal processing increases palatability and flavour, removes anti-nutritional factors, and improves nutrient bioavailability [7]. Thermal processing also leads to protein denaturation, peptide chain stretching, the disruption of conformational epitopes [8], and changes in spatial structure (i.e., transformation to a disordered state and/or aggregation) [9]. This behaviour can cause changes in protein properties and functions [10] and facilitate protein digestion and absorption, enzymolysis by digestive enzymes, and the release of active peptides. Watanabe, Kato, and Ayugase [11] found that coix seed protein fractions can reduce the glycaemic index of the pancreas in diseased mice. Coix seed prolamin (CSP) can also interfere with the IKK/NF-κB signalling pathway and regulate the expression of key genes, thereby contributing to the repair of pancreatic β-cells and insulin resistance [12]. We speculate that this may be due to the release and absorption of active peptides with hypoglycaemic effects in mice after the digestion of CSP. However, it is not yet known whether the active peptide with the ability to control blood glucose levels and regulate type two diabetes mellitus (T2DM) can still be released after the effect of processing on CSP. Furthermore, CSP contains extremely high levels of key amino acids, including proline, leucine, and alanine. Additionally, it contains the active peptide sequence signature that inhibits dipeptidyl peptidase-IV (DPP-IV), which is a key target for the treatment of T2DM. This protein accelerates the degradation of GLP-1, thereby affecting pancreatic beta cells and insulin secretion [13]. Therefore, CSP may be a potential source of DPP-IV inhibitory peptides after it is enzymatically cleaved during digestion. The newly released inhibitory peptides would reduce the risk of developing T2DM. To date, there have been no reports on DPP-IV inhibitory peptides derived from CSP.

The analysis of active peptides in food protein often involves the following three aspects: bioinformatics predictions, in silico simulations, and in vivo and in vitro experiments. Bioinformatics can be used to study the potential of proteins as sources of active peptides, the mechanisms by which they function, and the structure–function relationship in peptides [14]. Using in silico simulations to predict the results of protein digestion and the activity of the resulting peptide fragments saves time and money. Gangopadhyay et al. [15] used in silico tools to predict the possibility of the release of ACE-inhibiting peptides from barley protein, and then used in vitro experiments to confirm the reliability and accuracy of the predicted results. Dai et al. [16] used bioinformatics to evaluate the potential of sorghum protein as a precursor for DPP-IV inhibitory peptides. Currently, using bioinformatics to study the bioactive peptides released after the digestive enzymatic hydrolysis of coix seed proteins is uncommon.

Therefore, we used in silico techniques to assess the potential of CSP to act as a precursor of bioactive peptides by simulating its enzymatic digestion and predicting the activity and absorption of the resulting peptides. We also investigated the effects of two thermal processing methods on the amino acid composition and structure of CSP and its digestive products with DPP-IV inhibitory activity. This study provides a theoretical basis for the modification of CSP and the development of DPP-IV inhibitory peptides. It is also of importance for improving CSP applications in the food industry and the processing of coix seed products.

## 2. Materials and Methods

### 2.1. Materials and Reagents

Coix seeds (variety: small coix seed) were produced in Xingren, Guizhou, China. The coix seeds were purchased from Ganzhou Kangrui Agricultural Products Co. The electrophoretic reagent was purchased from Beijing Solarbio Technology Co., Ltd. (Beijing, China). The 8-anilino-1-naphthalenesulfonic acid (ANS), dithiothreitol (DTT), and other analytical reagents were all purchased from Sigma-Aldrich Chemical Co. (St. Louis, MO, USA). The DPP-IV (EC 3.4.14.5, 1VIAL) and Gly pro-p-nitroanilide were purchased from Sigma-Aldrich (St. Louis, MO, USA). The trichloroacetic acid and Tris–HCl buffer were purchased from Shanghai Macklin Biochemical Co., Ltd. (Shanghai, China). Unless otherwise noted, ultrapure water was used for all experiments.

### 2.2. Processing of Coix Seed and Preparation of Samples

Both the SF and BSF processes were conducted with reference to the Chinese Pharmacopoeia 2020 edition (Ch.P.2020) [17], and the product properties met the criteria specified in Ch.P.2020. After processing, the coix seeds were cooled and dried, and then broken up and sieved through an 80-mesh screen. The coix seed powder was then defatted using petroleum ether (*w*/*v* = 1:5) and dried to obtain defatted coix seed powder.

BSF processing method: Preheat the pan and sprinkle the bran evenly (10–15 kg of bran per 100 kg of coix seeds). Wait for the smoke to rise and then stir-fry the cleaned coix seeds with the bran. Stir-fry over medium heat until the coix seeds are dark yellow on the surface. Remove from the pan, sift the bran, and leave to cool.

SF processing method: Take about 25 kg of clean coix seeds and place in an automatic temperature control frying machine (XCYD-900, Hangzhou Jinzhu Machinery Co., Hangzhou, China). Stir-fry at 25 r/min and at a temperature of about 360 °C. Stir-fry until the coix seeds are yellow on the surface, and then remove and allow to cool.

### 2.3. In Silico Digestion and Bioactivity Prediction

The sequences of coix seed prolamins were obtained from the UniProt database [18] (Table A1 in Appendix A). Each protein sequence was digested in silico using trypsin (EC 3.4.21.4) and pepsin (3.4.23.1). The fragments were compared against those in the BIOPEP-UWM database [19], and the degree of hydrolysis and the sequences of the released peptides were predicted. The biological activity of each peptide was predicted using Peptide Ranker [20]. The DPP-IV inhibitory activity of each peptide was predicted using SwissTargetPrediction [21]. The absorption and transport of each peptide was predicted using ADMETlab [22]. The toxicity and sensitisation of each peptide were predicted using SVM (Swiss-Prot-based) [23] and AllerTOP [24], respectively.

### 2.4. CSP Extraction and Extraction Rate Measurement

The method of Liu et al. [13] was used with appropriate modifications. CSP extraction was performed by alcoholic extraction and water sedimentation. First, 80% ethanol, 1% DTT at a concentration of 1 mg/mL, and 1% NaAc (3 mol/L, pH = 6) were added to defatted coix seed powder, with an overall liquid-to-material ratio of 1:6. The samples were extracted by ultrasonication at 40 °C for 30 min and then centrifuged at 3500× *g* for 20 min. Deionised water was added in equal proportion to the supernatant, and the samples were then dialyzed at 4 °C for 24 h.

To the diluted extraction solution, 1 mL of basic copper solution and 4 mL of forinol solution were added, and then the solution was immediately shaken. Next, the solution was incubated in a water bath at 55 °C for 5 min. It was then transferred to an ice water bath for 10 min. Finally, the absorbance at 650 nm was measured to determine the protein content [25]. The prolamin extraction rate was calculated using the following formula:

$$\mathrm{Extraction}\;\mathrm{rate}\;\mathrm{of}\;\mathrm{prolamins}=\frac{\mathrm{Protein}\;\mathrm{content}\;\mathrm{in}\;\mathrm{extracts}}{\mathrm{Prolamins}\;\mathrm{content}\;\mathrm{in}\;\mathrm{coix}\;\mathrm{seed}}\times 100\%$$

### 2.5. Amino Acid Composition and Evaluation

The amino acid content was determined using the procedure listed in GB/T5009.124-2016 [26]. An appropriate amount of sample was added to 6 mol/L HCl and then hydrolysed in a constant temperature chamber at 110 °C for 22 h. Then, the sample was removed and cooled to 25 °C. The pH of the solution was adjusted to 2.2 using citrate buffer. This solution was then passed through a 0.22 μm filter membrane and bottled for testing.

The calculation of the amino acid score (AAS) was based on the Tilahun method, using the following formula [27].

$$\mathrm{AAS}=\frac{\mathrm{Amino}\;\mathrm{acid}\;\mathrm{content}}{\mathrm{Amino}\;\mathrm{acid}\;\mathrm{content}\;\mathrm{in}\;\mathrm{the}\;\mathrm{FAO}/\mathrm{WHO}\;\mathrm{scoring}\;\mathrm{scale}\;\mathrm{model}}\times 100$$

The protein efficiency ratio (PER) was calculated using the amino acid composition of CSP, which was determined based on the following three equations given by Mir, Riar, and Singh [28]. The PER values were calculated based on leucine, proline, tyrosine, methionine, and histidine content, as shown below. A PER value greater than 2 indicated that the protein had high nutritional value.

PER1 = −0.684 + 0.456 × Leu − 0.047 × Pro

PER2 = −0.468 + 0.454 × Leu − 0.105 × Tyr

PER3 = −1.816 + 0.435 × Met + 0.78 × Leu + 0.211 × His − 0.944 × Tyr

### 2.6. Protein Structure Determination

#### 2.6.1. Determination of Surface Hydrophobicity

The samples were dissolved in 80% ethanol to a concentration of 1 mg/mL. The resulting concentration gradient dilution was mixed with 8 mmol/L ANS at a volume ratio of 100:1 and the fluorescence intensity of the solution was measured after 3 min. The excitation wavelength was 390 nm and the emission wavelength was 468 nm. The initial slope of the fitted curve was calculated as the hydrophobic value of the sample surface, H_0_, using the fluorescence intensity as the dependent variable and the concentration of CSP as the independent variable [29].

#### 2.6.2. Fourier-Transform IR Spectroscopy

The infrared spectra (Tensorii FTIR) were determined using methods consistent with those described by Yasar, Tosun, and Sonmez [30]. The amide I band in the spectrogram was deconvoluted, fitted, and analysed using Gaussian curve fitting in Peakfit 4.12 software. The relative content of its main characteristic peaks (amide I band, 1700~1600 cm^−1^) was determined (Table 1), and thus the trend of the protein secondary structure was explored.

#### 2.6.3. Raman Spectroscopy

The samples were measured using Raman spectrometry. The spectral conditions were: excitation wavelength: 785 nm; emission power: 300 mW; spectral range of measurement: from 500 to 2000 cm^−1^. The Raman spectra of the measured samples were plotted after the accumulation of the signals and calculation of the mean values. The baseline correction and attribution of the peaks were performed using WiRE 5.2 software (RENISHAW Co., Hong Kong, China). The spectra were fitted using Origin 2022 software(OriginLab Co., Hampton, MA, USA).

#### 2.6.4. Fluorescence Spectrum (FS) Measurement

The structures of CSP were measured using the FS measurement method described by Zhang et al. [29], with minor modifications. The protein concentration was fixed at 10.0 mg/mL. The excitation wavelength was fixed at 280 nm and the emission wavelength at 290–450 nm (Fluoromax-4cp fluorescence spectrometer, 25 °C). The slit width for both excitation and emission was set at 5 nm. A quartz cuvette with four-sided light transmission and a thickness of 10 mm was used.

#### 2.6.5. Determination of Particle Size

The particle size distribution of the CSP was determined using a PALS-laser particle size analyser. The sample concentration was 0.5 mg/mL, the solvent was 80% ethanol, and the injection volume was 1 mL. The measurement was performed at room temperature (25 °C) [31].

#### 2.6.6. SDS-PAGE Analysis

The CSP sample concentration was 0.5 mg/mL. The samples were denatured in a boiling water bath before loading (10 μL/well). A 5% stacking gel and a 12% separating gel were used. Electrophoresis was performed at 80–120 V. The gels were photographed after staining and decolourisation [32]. The formulation and timing of the colourant and decolouring agent are specified below.

Colourant: 2.5 g of Coomassie Brilliant Blue R250, 250 mL of 95% ethanol, 80 mL of glacial acetic acid, fixed volume of distilled water to 1000 mL. Decolouring agent: 80 mL of glacial acetic acid, 250 mL of 95% ethanol, fixed volume of distilled water to 1000 mL. The gel needs to be coloured for 2 h and decoloured four times for 1.5 h each.

### 2.7. Determination of the Polypeptide Content

A mixture of hydrolysates and 10% (*w*/*v*) TCA was vortexed, allowed to stand for 10 min, and then centrifuged at 4000 rpm for 15 min. The supernatant was diluted with a 5% TCA solution for further use. The sample and biuret reagent were mixed in a 3:2 (*v*/*v*) ratio, allowed to stand for 10 min, centrifuged (3000× *g*, 15 min), and the absorbance of the solution was measured at 540 nm. The peptide content of the hydrolysates was calculated using a standard curve (y = 0.0721x + 0.069, R^2^ = 0.9987) [33].

### 2.8. Determination of DPP-IV Enzyme Inhibitory Activity (In Vitro Substrate Chemical Method)

After adding 40 μL of the sample and 50 μL of the substrate Gly-Pro-p-nitroanilide (0.4 mmol/L) to a 96-well plate and incubating it for 10 min at 37 °C, 10 μL of DPP-IV (0.05 units/mL) was added to initiate the reaction at 37 °C. After 60 min, the absorbance value was measured at 405 nm [16,34]. The sample blank group (Asb) contained Tris–HCl buffer instead of DPP-IV; the control group (Ac) contained Tris–HCl buffer instead of the sample; the control blank group (Acb) contained Tris–HCl buffer instead of the sample and DPP-IV.

(1) $$\mathrm{DPP}-\mathrm{IV}(\%)=1-\frac{\mathrm{As}-\mathrm{Asb}}{\mathrm{Ac}-\mathrm{Acb}}\times 100\%$$

### 2.9. In Vitro Simulated Digestion of CSP

The effects of simulated gastric and intestinal digestion on the peptide content and DPP-IV inhibitory activity of CSP before and after processing were examined, as described by Wang et al. [35]. A 1 g prolamin sample was loaded into a digestion flask and digested in a water bath shaker at 37 °C and 200 rpm. The pH was adjusted to 3.0, using 1 mol/mL HCL. The 10 mL of artificial gastric juice (pH 3.0) was added, and the digestion reaction was carried out for 2 h to simulate the gastric digestion phase. The pH of the digestion system was adjusted to 7.0 with 1 mol/mL NaOH. Then, 15 mL of an artificial intestine solution (pH 7.0) was added to the digestion system and allowed to react for 2 h to simulate the small intestine digestion phase, followed by 15 min of enzyme inactivation in a boiling water bath. The peptide content and DPP-IV inhibitory activity of the digests were measured at various stages of simulated digestion in vitro.

### 2.10. Statistical Analysis

The results are expressed as the mean ± standard deviation (X ± S). SPSS software was used for the univariate analysis of variance (ANOVA) and Duncan’s test (*p* < 0.05), and differences between groups were compared using the t-test. Origin software was used for plotting, and Peakfit software was used for structural analysis. Image J software was used for the grey-scale analysis of the protein bands.

## 3. Results and Discussion

### 3.1. Predicted Release and Analysis of DPP-IV Inhibitory Peptide after Gastrointestinal Digestion of CSP

#### 3.1.1. In Silico Bioactivity Prediction

**Figure A1:**
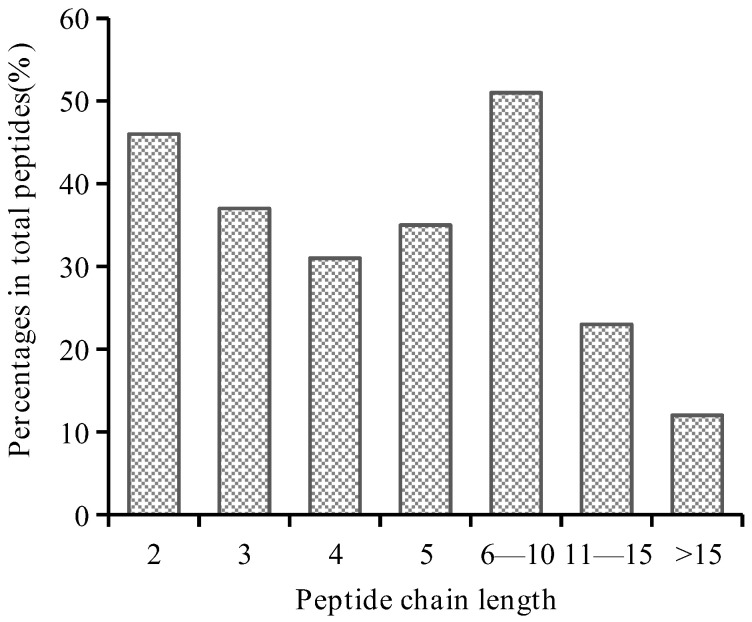
Chain length of the peptide after CSP digestion was predicted in silico.

Six full sequences of CSP were retrieved from the UniProt database. Most of the CSP sequences belonged to one of three types: α-coix, β-coix, and γ-coix. In the in silico simulated enzymatic digestion, the degree of hydrolysis ranged from 11.22% to 26.21%, and a total of 235 peptides were obtained. Of these, 63.40% were peptides with chain length < 5 (Table A1 in Appendix A and Figure A1 in Appendix A). We identified 47 CSP-derived bioactive peptides in silico (Table 2). We considered a peptide to be biologically active if Peptide Ranker predicted a value > 0.5 [36]. Among these peptides, 64.58% had potential DPP-IV inhibitory activity (Table 1). Five peptides (PAL > QQPL > PL > PSL > SSPL) showed the highest probability of having DPP-IV inhibitory activity. The DPP-IV inhibitory activity is thought to be related to the type and sequence of amino acid residues in the peptide chain, hydrophobicity, and functional groups. The higher the content of hydrophobic amino acids in the active peptide, the greater the potential for the inhibition of DPP-IV activity and the stronger the activity. There is a similar relationship between peptide activity and the presence of hydrophobic amino acids in the N-terminal region. The inhibition rate of the active peptide was also higher when Pro was present at the penultimate position of the amino acid sequence or at the C-terminus [37]. The active peptides that were screened satisfied these criteria.

#### 3.1.2. Prediction of Digestion and Absorption, Toxicity, and Allergenicity of Active Peptides

Bioactive peptides must survive degradation by gastrointestinal digestion, reach their target intact, and remain bioavailable to exert a beneficial physiological effect. Hence, the peptides were analysed in silico to predict their absorption, transport, toxicity, and allergenicity (Table 1). None of the peptides were toxic, but allergenicity was possible. The Caco-2 permeability of the active peptides was moderate (optimal: higher than −5.15 Log unit), and 85.11% of the active peptides had improved MDCK permeability (low permeability: <2 × 10^−6^ cm/s; medium permeability: 2–20 × 10^−6^ cm/s; high passive permeability: >20 × 10^−6^ cm/s) [22]. These results indicated that the active peptides released after CSP digestion were more effectively absorbed and transported in normal cells than they were in cancer cells. The percent human intestinal absorption (%HIA) was 42.55%, indicating that the active peptides had high intestinal absorption (+ (HIA < 30%); − (HIA ≥ 30%); +, − with increasing degree) [22]. This was especially true for peptide chains containing Leu and Phe at the carboxyl terminus. In addition, 87.23% of the peptides were predicted to penetrate the blood–brain barrier. Plasma protein binding (PPB) and the fraction unbound in plasma (Fu) capacity was expressed as the ability to bind to plasma proteins. The active peptides identified had a relatively low PPB and high Fu, suggesting the presence of a good therapeutic index. The comprehensive prediction results indicate that the transport and absorption ability of CSP-derived active peptides (including DPP-IV inhibitory peptides) and their ability to bind proteins were excellent, providing theoretical support for them to be used as functional foods and drugs.

### 3.2. Effect of Processing Method on CSP Extraction Rate

**Figure 1:**
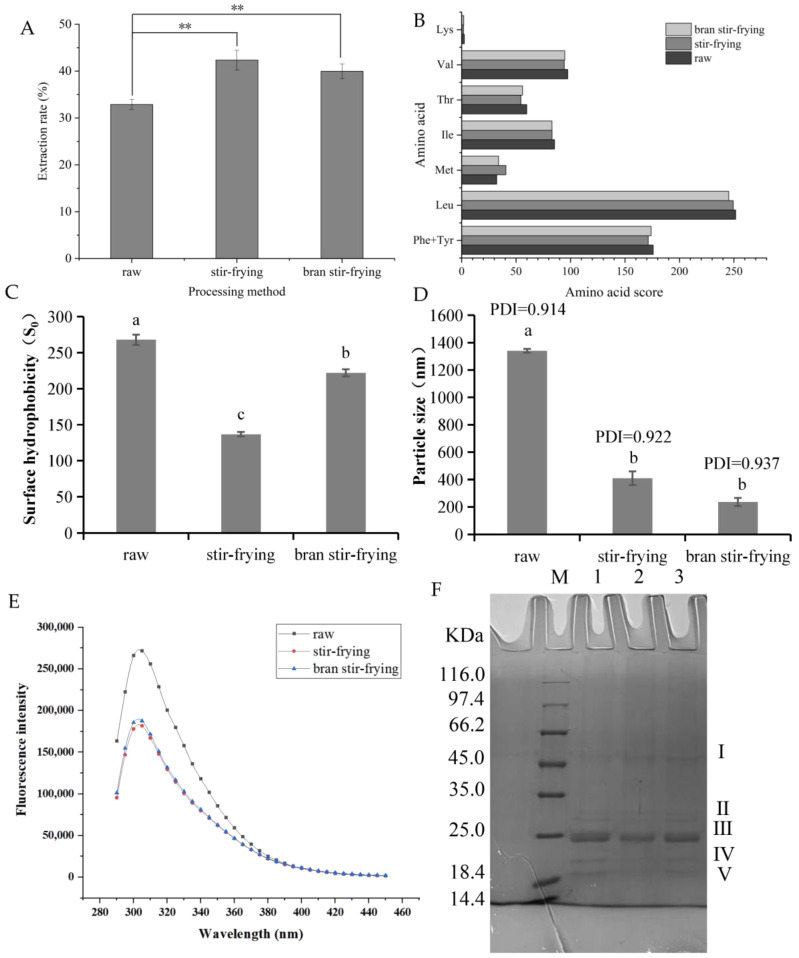
(**A**) Extraction rates of prolamin from coix seed under different processing methods. ** indicates a significant difference (*p* < 0.01). (**B**) Evaluation of amino acid content of coix seed. Note: Adult requirement score according to WHO/FAO amino acid pattern. (**C**) Surface hydrophobicity of coix seed prolamin with different processing. Different letters (a–c) indicate significant difference between the mean values of different samples (*p* < 0.05). (**D**) Particle size of coix seed prolamin at different thermal processing. Different letters (a,b) indicate significant difference between the mean values of different samples (*p* < 0.05). (**E**) Two-dimensional fluorescence spectrogram at different processing of coix seed prolamin. (**F**) SDS-PAGE electrophoresis diagram of coix seed prolamin at different heat treatment methods. M lane: marker; 1 lane: raw; 2 lanes: bran stir-frying; 3 lanes: stir-frying.

The unique solubility and strong self-assembly properties of CSP have made it difficult to isolate and extract and have limited enzymolysis-based studies. In this study, the CSP extraction efficiency significantly increased by 28.83% and 21.59% after SF and BSF, respectively (Figure 1A). Three possible reasons for this are (1) thermal processing may disrupt the cellular structure of coix seeds, making their prolamin more easily dispersed; (2) heat treatment may dissociate nutrients such as starch and oil that are tightly bound to prolamin in coix seeds, increasing the protein exposure to extraction media; and (3) processing may lead to the dissociation of prolamin, in which the residual non-covalent bonds within or between the molecules are broken after a reducing agent has perturbed them, causing the prolamin polymer spatial structure to become disordered [25]. As shown in Figure 1, the extraction was slightly better for SF than for BSF, but the difference between the two was not statistically significant. This may be due to the higher temperature the smoked coix seeds attained after the bran had been smoked. A slow fire was used for SF, and so the coix seeds did not reach a high temperature. High-temperature processing causes the loose polypeptide chains resulting from prolamin denaturation to recombine into tighter polymers. These polymers form after the regeneration of disulphide and hydrophobic bonds, and they impede interactions between the solvent and the CSP [38].

### 3.3. Amino Acid Composition, Evaluation, and Analysis of CSP after Different Processing Methods

Coix seed contains high-quality plant proteins. The amino acid composition of a protein is one of its main chemical properties, and it is also an important indicator for evaluating biological value and quality. Table 3 shows the sixteen amino acids found in CSP. Of the total amino acids (TAAs), nine are non-essential amino acids (NEAAs) and seven are essential amino acids (EAAs). No Trp was detected, which was consistent with the results of Liu et al. [12]. The mean value for the EAA/TAA ratio of CSP was 34.397%, and the mean value for the EAA/NEAA ratio was 52.081%, which is close to the ideal protein pattern (E/T > 40% and E/N > 60%) proposed by FAO/WHO (Table 3). The EAA content of CSP also decreased significantly after processing. Moreover, it can be seen from the amino acid score plot in Figure 1B that Leu had the highest amino acid score in CSP, followed by Phe + Tyr, whereas Lys and Met were the first and second limiting amino acids, respectively. The limiting amino acid scores increased after SF and BSF, whereas the AAS of amino acids with higher initial scores decreased, but not significantly. In addition, as noted in Table 3, the PER value of CSP ranged from 6.76 to 8.183. A PER value greater than 2 indicates good protein quality. Although the PER value of CSP decreased after processing, it was still higher than those of cowpea, peanut, and potato proteins [28]. Coix seed protein also contains a high percentage (75.326%) of medicinal amino acids, which is the main reason for its multiple physiological effects.

As can be seen in Table 3, the content of Glu, Leu, Ala, Pro, Phe, and Asp in CSP was higher than that in general coarse cereals and grains. The Leu content is even higher than that of *Cordyceps*, suggesting it could be a good supplement for cereal products containing low amounts of Leu. Leu has been reported to improve glucose uptake by skeletal muscle, alleviate insulin resistance, and lower cholesterol levels [39]. It was also found that CSP contained a high proportion of hydrophobic amino acids. It has been shown that protein sources with higher contents of Pro, Ala, and other hydrophobic amino acids were more likely to be found in DPP-IV inhibitory peptides that regulate T2DM and restore β islet cell physiological activity [37]. The results shown in Table 3 show the effect of heat processing treatments on the amino acid content of coix seed protein. However, there were differences in the types of amino acids affected by the different heat treatments, which caused different trends in the results. The content of all 11 amino acids tended to decrease after BSF, which could be due to irreversible protein breakdown. This could also be caused by severe damage to or the loss of amino acids during processing [40]. There was a greater increase in Asp, Ser, and Ala content after SF than after BSF. Mariotti [41] found that unheated or lower heating temperatures caused amino acids to be protected in the food matrix, thus affecting their bioavailability. However, high heating temperatures caused amino acid liberation and elevated levels of free amino acids [41]. This could explain the trends in the present study. Both thermal processes resulted in increased levels of Met and Gly.

In terms of flavour amino acid composition, raw coix seed had a greater proportion of bitter amino acids, followed by sweet and fresh amino acids. The TAV values of the flavourful amino acids in coix seed were all greater than 1. This means they make a substantial contribution to the overall flavour of coix seed, with multiple flavour-presenting amino acids acting in synergy to form its unique flavour. After processing, the B/T showed a slow decrease, S/T showed a slow increase, and F/T did not change significantly. The BSF process had a greater impact on the TAV values of the flavourful amino acids of barley, and the TAV values of the three main categories of flavouring amino acids were reduced after this process. It is possible that after processing, some free amino acids were produced and some were degraded, thereby forming different volatile compounds that reduced the bitterness of the coix seed itself and increased its aroma and flavour. In summary, the amino acids of raw and cooked coix seed were somewhat different, suggesting that processing had altered the flavour and amino acid availability to some extent.

### 3.4. Effect of Different Processing Methods on Prolamin Structure

#### 3.4.1. Surface Hydrophobicity Analysis

Surface hydrophobicity is an important parameter for measuring the strength of intermolecular interactions. It is not only related to processing methods and conditions, but also to the inherent nature of the protein. The results in Figure 1C show that the surface hydrophobicity of the prolamin was significantly reduced after SF and BSF. This could be because thermally disordered peptide chains formed at high temperatures experience hydrophobic interactions between newly exposed residues during cooling. This can cause non-native inter-subunit interactions and the hydrophobic aggregation of proteins, which can lead to conformational changes that further affect the distribution and density of amino acid residues and the number of hydrophobic holds on the surface of protein molecules. Thermal processing also caused the deamination of exposed Asn and Gln residues on the protein surface, causing hydrophobic amino acid residues to become buried and a reduction in surface hydrophobicity [42]. The results were consistent with those in [43].

Because BSF requires the addition of bran and relies on rapid frying in smoking bran for a shorter time compared to normal SF, hydrophobic amino acids and groups may not be completely masked during this process. If they are not, the surface hydrophobicity of the prolamin would be higher after BSF than after SF. Reduced hydrophobic interaction between proteins would increase the digestibility of proteins in vivo, indicating that processing can make it easier for the body to digest, absorb, and utilise CSP.

#### 3.4.2. Particle Size

The particle size of CSP after different processing methods is shown in Figure 1D. The particle size of the prolamin was significantly reduced after processing. This means that the spherical structure of the prolamin was disrupted during thermal processing, and that it formed an irregular polymer, which had smaller particles and was better dispersed in the solvent. Nevertheless, the effect of the different processing methods on the particle size of prolamins was not statistically significant. Heat treatment can lead to cross-linking or aggregation between protein molecules, and the degree of aggregation increases during thermal processing [44]. Zhao et al. [45] found that after thermally processing the 11S monomer, the globulins easily formed covalent aggregates to increase particle size. The reason for the difference between these results and the ones reported in this paper may be that the prolamin existed as a higher-order self-assembled structure and the initial particle size was larger than that of ordinary monomeric storage proteins. Although protein aggregation occurred after heat treatment, the particle size was still lower than the protein particle size before heat processing.

#### 3.4.3. Sodium Dodecyl Sulphate-Polyacrylamide Gel Electrophoresis

**Figure A2:**
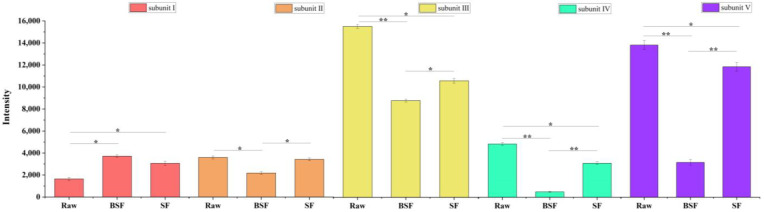
Grey-scale analysis of CSP protein bands, * *p* < 0.05, ** *p* < 0.01.

The molecular masses of the CSP before and after processing were characterised using sodium dodecyl sulphate–polyacrylamide gel electrophoresis (Figure 1F and Figure A2). The molecular weight of the unprocessed prolamin was mainly distributed between 18.4 and 35.0 kDa, and the α-prolamin content was the highest. The II (30.22 kDa), III (23.35 kDa), IV (20.18 kDa), and V (19.38 kDa) subunit bands in the CSP gradually became lighter after processing. The two bands with smaller molecular weights eventually disappeared completely under BSF processing conditions. This could be due to the degradation and conversion of the protein to smaller molecular weight peptides after thermal processing. These peptides would migrate rapidly in the isolate gel, and their presence would not be reflected in the 12% isolate gel. Both bands contained γ-prolamins [46], which have a relatively low thermal stability and easily decompose. The formation of a larger molecular weight subunit band I (52.52 kDa) after SF may have been due to the high temperature-induced aggregation of the soluble components of the prolamin molecule [47]. This further explains why the protein particle size was reduced, but different after both SF and BSF. Voci, Fresta, and Crosco [48] found that the γ-prolamin of gliadins ranged from 55 to 70 kDa. Thus, it is presumable that the polymer formed after SF was similar in structure to the γ-prolamin of gliadins.

#### 3.4.4. Spectral Analysis

We used endogenous fluorescence spectroscopy to study the molecular spatial conformation of CSP. Tyr (348 nm), Tyr (303 nm), and Phe (282 nm) are the main amino acid residues that cause endogenous fluorescence in the protein. The maximum emission wavelength of CSP was found to be 305 nm, and no strong fluorescence peaks were observed near 348 nm or 282 nm. Trp is the amino acid residue with the highest fluorescence intensity, followed by Tyr and Phe. However, the amino acid composition analysis confirmed that the amount of Trp in CSP was extremely low. Furthermore, Phe was not excited under most experimental conditions, so the emission of Phe was rarely observed. Therefore, the main amino acid that caused fluorescence in CSP was Tyr (Figure 1E). The fluorescence intensity of coix seed decreased significantly after thermal processing, and the peak shape was significantly altered and shifted. This was probably due to prolamin structure disruption and polarity changes in the microenvironment near the tyrosine residues, which would result in fluorescence quenching. In addition, several studies have revealed that heat treatment promoted the interaction between proteins and active small molecules. This can increase the energy transfer from amino acid residues and accelerate protein fluorescence quenching [29,49,50].

**Figure 2:**
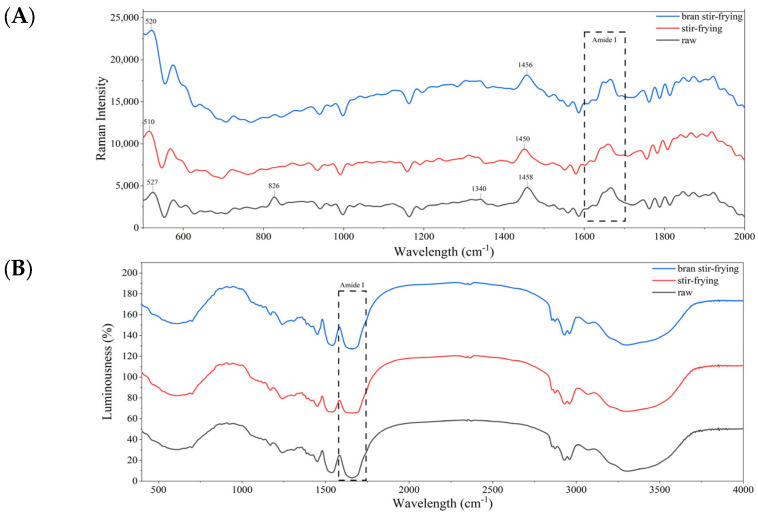
Raman spectroscopy (**A**) and Fourier-transform infrared spectroscopy (**B**) of coix seed prolamin under different processing.

The secondary structure of a protein is closely related to the type of chemical bonds between the protein molecules. Raman spectroscopy and FTIR are powerful tools for studying the types of chemical bonding in molecules. The FTIR and Raman spectra of coix seed after different processing methods are shown in Figure 2. After processing, the absorption peak at 826 cm^−1^ disappeared, and the remaining peak was in the Fermi resonance variation range of tyrosine in Raman spectroscopy. The Fermi resonance of tyrosine resulted in characteristic peaks around 850 cm^−1^ and 830 cm^−1^ depending on the side chain microenvironment. When the intensity ratio I850/I830 was ≥1, the tyrosine was exposed, but when the intensity ratio I850/I830 was <1, the tyrosine was buried [51]. Therefore, processing could lead to tyrosine transitioning from the embedded to exposed state, as the peak ratio was closely related to the extent of the heat processing (raw 0.77 < 1, bran-fried 0.88 < 1, fried 1.07 > 1). Another characteristic Raman peak was attributable to the disulphide bond characteristic spectrum at 500–550 cm^−1^ with three configurations: g-g-g (510 cm^−1^), g-g-t (516–530 cm^−1^), and t-g-t (531–545 cm^−1^) [51]. After processing, the characteristic peak gradually redshifted. When the temperature or intensity of the processing method is more violent, the characteristic band of the CSP disulphide bond will change from the g-g-t configuration to the more stable g-g-g configuration.

The protein secondary structure distribution estimated for all samples according to the thermal processing method of the coix seed followed the order β-sheet > α-helix > β-turn > random coil (Table 4). However, SF and BSF both had a negative effect on the β-fold and β-turned structure of CSP, confirming that the extent to which CSP molecules correctly fold is reduced by thermal processing. The β-folded structure is positively correlated with the level of protein molecule aggregation [52]. Additionally, it was confirmed that thermal processing led to a decrease in the aggregation level of CSP, which was consistent with the particle size results. However, processing positively affected α-helix and random coil structures, indicating that heat treatment could disrupt the original rigid structure of prolamin. This disruption increases the protein flexibility and changes its conformation from ordered to disordered. Moreover, heat treatment may perturb the hydrogen bonds and weaken the non-covalent interactions between protein molecules, resulting in a broader distribution of molecular free energy [53].

### 3.5. Effect of Processing on the Peptide Content and DPP-IV Inhibitory Activity of CSP Gastrointestinal Digestion Products

**Figure 3:**
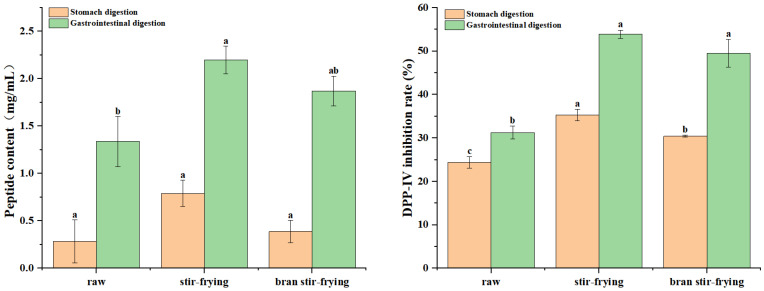
Effect of processing on peptide content and DPP-IV inhibitory activity of the digestive hydrolysate from coix seed prolamins. Different letters (a–c) indicate significant difference between the mean values of different samples (*p* < 0.05).

The predicted results of enzymatic digestion by computer simulation confirmed the possible DPP-IV inhibitory activity of CSP-derived peptides (Table 2). The in vitro digestion results showed that the peptide content of the gastric and gastrointestinal digestion products of CSP was 0.28 mg/mL and 1.15 mg/mL, respectively, and the DPP-IV inhibitory activity was 24.38% and 31.27%, respectively (Figure 3). After pepsin cleavage, CSP could release peptides with DPP-IV inhibitory activity. Trypsin digestion in the intestine recognises the cleavage site of the protein, which releases more DPP-IV inhibitory peptides and significantly enhances the inhibitory activity.

In addition, analysing the effects of both SF and BSF on the structure and functionality of CSP revealed that processing significantly changed the conformation of the prolamins. It has been reported that the gastrointestinal digestion of proteins is mostly driven by the protein matrix structure and composition and the available contact area between the protein substrate and digestive enzymes [43]. As shown in Figure 3, the peptide content of CSP gastric digestive products did not change significantly after SF or BSF processing, and the DPP-IV inhibition activity increased significantly. The peptide content and DPP-IV inhibitory activity of the CSP gastrointestinal digestive products increased significantly by 19.89% and 36.84%, respectively, after BSF processing. The peptide content and DPP-IV inhibitory activity of CSP gastrointestinal digestive products increased significantly by 30.91% and 42.02%, respectively, after SF processing. In summary, the processed CSP may be better suited to human digestion, absorption, and utilisation. In addition, the DPP-IV inhibitory effect after SF processing is more significant.

## 4. Conclusions

Whether CSP digestion can result in peptides that precisely modulate DPP-IV has not been previously reported. Furthermore, it is unknown whether processing can affect the structure of CSP and accelerate the release of DPP-IV inhibitory peptides. Therefore, we predicted and analysed the activity and absorption and transport capacity of the peptides by simulating the digestion of CSP in silico and found that 64.58% of the resulting bioactive peptides had the ability to inhibit DPP-IV. These peptides also had good absorption and transport capacity. The effect of processing on the structure of CSP and its DPP-IV inhibitory activity after gastrointestinal digestion was also investigated. We found that both SF and BSF processing could alter the secondary structure of CSP, accelerate the release of DPP-IV inhibitory peptide, and enhance the peptide content and inhibitory activity of digested products. We hope that this study improves the current understanding of the added value of coix seed and provides a theoretical basis for the development of coix seed-derived hypoglycaemic products.

## Figures and Tables

**Table 1:** Attribution basis of each peak position.

Structure	Wavelength
β-sheet	1615~1637 cm^−1^, 1682~1700 cm^−1^ and 1230~1240 cm^−1^
α-helix	1646~1664 cm^−1^ and 1265~1300 cm^−1^
Random coil	1637~1645 cm^−1^ and 1240~1260 cm^−1^
β-turn	1664~1681 cm^−1^ and 1305 cm^−1^

**Table 2:** Prediction of molecular weight, allergenic, toxicity, DPP-IV inhibition activity, Caco-2 permeability, MDCK permeability, Human Intestinal Absorption (HIA), plasma protein binding (PPB), blood–brain barrier penetration (BBB penetration), and the fraction unbound in plasma (Fu) of biological peptide from coix seed prolamin digestion by in silico prediction.

Bioactive Peptide	Molecular Weight (Da)	Biological Activity	DPP-IV Inhibits Activity Probability	Allergenic	Absorption			Transport		
					Caco-2 Permeability	MDCK Permeability	HIA	PPB	BBB Penetration	Fu
WGF	408.18	0.997	0.114	*	−6.147	8.90 × 10^−6^	---	67.13%	-	41.60%
PF	262.13	0.993	0.120	*	−5.911	1.70 × 10^−5^	++	16.74%	-	75.73%
PPF	359.18	0.989	0.117	*	−5.979	2.40 × 10^−5^	+++	22.82%	--	73.78%
AF	236.12	0.973	0.049	#	−6.109	0.0011	---	15.81%	++	75.64%
QMPF	521.23	0.971	0.103	*	−6.277	9.60 × 10^−6^	++	17.36%	--	69.34%
IF	278.16	0.949	0.101	#	−5.891	0.00014	---	50.43%	++	43.75%
SF	252.11	0.949	NA	#	−6.168	0.0034	---	12.01%	++	80.45%
VMPF	492.24	0.941	0.120	*	−6.387	1.40 × 10^−5^	++	33.07%	---	57.91%
PSMCGL	606.25	0.900	NA	*	−6.928	2.50 × 10^−6^	+++	15.43%	---	77.17%
ML	262.14	0.895	0.049	#	−5.803	2.00 × 10^−5^	---	9.38%	--	81.31%
PCNPL	542.25	0.858	0.126	*	−6.576	1.30 × 10^−5^	+++	9.90%	---	79.71%
MAPQIF	705.35	0.834	0.060	#	−6.385	2.90 × 10^−5^	+++	39.54%	---	52.31%
PL	228.15	0.811	0.425	*	−5.667	8.00 × 10^−5^	-	7.64%	--	84.36%
SWQQPIVGGVGF	1273.65	0.768	-	*	−5.645	2.20 × 10^−5^	++	55.93%	---	40.33%
PPVSAIGF	786.43	0.766	NA	#	−6.928	2.30 × 10^−5^	+++	37.20%	---	56.18%
QMMMQL	780.33	0.758	0.050	#	−6.353	7.50 × 10^−6^	---	18.75%	---	65.28%
QQF	421.2	0.718	0.106	#	−5.960	0.00038	---	12.74%	-	73.26%
MNPAL	544.27	0.710	0.126	*	−6.605	2.00 × 10^−5^	-	8.99%	---	80.78%
QQALLGGALF	1016.57	0.689	-	#	−6.173	5.10 × 10^−5^	+	22.95%	---	50.89%
MAL	333.17	0.681	0.119	*	−6.128	2.00 × 10^−5^	---	7.45%	--	81.85%
AAAF	378.19	0.677	0.113	#	−6.593	0.00081	---	12.58%	-	75.18%
AAASPAAF	704.35	0.665	0.020	*	−7.140	0.00065	+++	17.31%	---	66.54%
PSNPL	526.28	0.656	0.142	*	−6.617	0.00026	++	13.12%	---	85.01%
QQCNPL	701.32	0.646	0.042	*	−6.271	0.00014	--	9.44%	---	79.38%
PHVSAIGF	826.43	0.638	NA	#	−6.730	2.70 × 10^−6^	+++	35.05%	---	54.75%
PAL	299.18	0.632	0.724	#	−6.019	0.00017	++	9.22%	--	84.24%
YPQAMANIAAF	1195.57	0.627	-	*	−6.449	2.80 × 10^−5^	+++	44.34%	---	39.59%
QPYR	562.29	0.616	NA	*	−6.666	0.00014	++	12.15%	--	71.84%
QPQCSCSPVAVPYYAQQR	2023.92	0.613	-	*	−6.751	3.00 × 10^−5^	+++	36.26%	---	35.62%
PSYCGTTPSCAVSAAIPPYY	2046.91	0.610	-	*	−8.250	3.80 × 10^−6^	+++	60.25%	---	30.74%
NCHEF	648.23	0.596	NA	#	−6.747	7.40 × 10^−6^	---	19.09%	--	72.76%
AGL	259.15	0.593	0.153	#	−6.478	0.0024	---	4.47%	+	87.34%
YPQAL	590.31	0.586	0.082	*	−6.457	0.00036	++	22.71%	---	68.21%
APTAAIIPR	908.54	0.584	-	*	−7.028	8.70 × 10^−5^	+++	28.99%	---	58.13%
QPYSL	606.3	0.567	0.066	#	−6.680	0.0006	++	16.25%	--	73.57%
YPCAEYL	857.36	0.564	NA	#	−7.080	7.10 × 10^−6^	+++	42.10%	---	48.35%
SSPL	402.21	0.550	0.362	*	−6.599	0.005	+	9.00%	--	88.11%
PSL	315.18	0.540	0.380	#	−6.222	0.001	++	9.61%	--	87.55%
NPAASCQQPIVGAAL	1438.72	0.535	-	#	−6.761	0.00012	+++	21.70%	---	53.67%
QGL	316.17	0.533	0.254	*	−6.422	0.0011	---	4.61%	+	85.41%
ASNPL	500.26	0.525	0.119	#	−6.894	0.0012	--	8.17%	--	86.93%
QQPL	484.26	0.523	0.469	*	−6.037	0.00036	--	8.26%	--	82.93%
QQHQPPPPQK	1183.61	0.518	-	*	−5.456	8.70 × 10^−5^	+++	25.09%	---	61.34%
AAANPAAYL	860.44	0.515	NA	*	−7.132	0.00015	++	22.84%	---	60.16%
CSYSYYSGNSNL	1356.53	0.507	-	*	−8.364	0.00012	-	27.60%	---	54.05%
VNF	378.19	0.505	0.113	*	−6.443	0.00067	---	10.57%	+	73.09%
AQQPL	555.3	0.505	0.297	*	−6.276	0.00029	--	8.93%	--	81.48%

Note: S—serine, E—glutamic acid, H—histidine, G—glycine, R—arginine, T—threonine, A—alanine, P—proline, C—cysteine, Y—tyrosine, V—valine, M—methionine, K—lysine, I—isoleucine, L—leucine, F—phenylalanine, N—aspartate, Q—glutamine. “*” means toxic or allergenic; “#” means non-toxic or non-allergenic; Genotoxic Carcinogenicity includes carcinogenicity or mutagenicity; “-” represents appropriate, “+” represents inappropriate, and the number of +/- represents extent.

**Table 3:** Content of essential amino acids in coix seed samples and comparative nutritional profile.

Essential Amino Acids	Amino Acid Content (g/100 g)			Non-Essential Amino Acids	Amino Acid Content (g/100 g)		
	Raw	Stir-Frying	Bran Stir-Frying		Raw	Stir-Frying	Bran Stir-Frying
Thr ^#^	2.39 ± 0.02 ^a^	2.18 ± 0.00 ^b^	2.24 ± 0.01 ^b^	Asp *^##^	5.98 ± 0.04 ^a^	5.99 ± 0.01 ^a^	5.89 ± 0.02 ^b^
Val **	4.87 ± 0.01 ^a^	4.71 ± 0.01 ^b^	4.74 ± 0.02 ^b^	Tyr ^##^	4.81 ± 0.03 ^a^	4.73 ± 0.02 ^b^	4.76 ± 0.03 ^b^
Met **^##^	1.13 ± 0.02 ^b^	1.42 ± 0.02 ^a^	1.19 ± 0.03 ^b^	Ser ^#^	4.62 ± 0.11 ^a^	4.68 ± 0.02 ^a^	4.54 ± 0.07 ^b^
Ile **	3.41 ± 0.01 ^a^	3.32 ± 0.02 ^ab^	3.32 ± 0.03 ^b^	Glu *^##^	**27.29 ± 0.53 ^a^**	**27.10 ± 0.10 ^a^**	**26.64 ± 0.68 ^a^**
Leu **^##^	**17.62 ± 0.05 ^a^**	**17.46 ± 0.03 ^a^**	**17.17 ± 0.06 ^b^**	Gly ^#^	0.57 ± 0.05 ^bc^	0.70 ± 0.04 ^a^	0.61 ± 0.02 ^b^
Phe **^##^	5.75 ± 0.01 ^a^	5.55 ± 0.02 ^a^	5.68 ± 0.05 ^a^	**Ala ^#^** **^##^**	**12.38 ± 0.10 ^b^**	**12.50 ± 0.02 ^a^**	**12.19 ± 0.03 ^b^**
Lys ^##^	0.14 ± 0.00 ^a^	0.10 ± 0.00 ^c^	0.11 ± 0.01 ^b^	His **	1.12 ± 0.07 ^a^	1.09 ± 0.00 ^b^	1.08 ± 0.02 ^b^
Trp *	0	0	0	Arg **^##^	2.23 ± 0.02 ^a^	2.23 ± 0.01 ^a^	2.15 ± 0.00 ^b^
TAA	102.66 ± 0.03 ^a^	101.96 ± 0.09 ^b^	100.52 ± 0.09 ^c^	**Pro**	**8.34 ± 0.04 ^a^**	**8.20 ± 0.02 ^b^**	**8.22 ± 0.01 ^b^**
EAA	35.31 ± 0.02 ^a^	34.74 ± 0.02 ^b^	34.44 ± 0.04 ^b^	EAA/TAA (%)	34.40 ± 0.08 ^a^	34.07 ± 0.03 ^a^	34.27 ± 0.09 ^a^
NEAA	67.345 ± 0.08 ^a^	67.22 ± 0.05 ^a^	66.08 ± 0.06 ^b^	EAA/NEAA (%)	52.43 ± 0.06 ^a^	51.68 ± 0.05 ^b^	52.12 ± 0.15 ^a^
F	5.98 ± 0.01 ^a^	5.99 ± 0.02 ^a^	5.89 ± 0.01 ^a^	F/T (%)	5.83 ± 0.01 ^c^	5.88 ± 0.02 ^a^	5.86 ± 0.01 ^b^
B	36.14 ± 0.07 ^a^	35.78 ± 0.03 ^b^	35.33 ± 0.03 ^b^	B/T (%)	35.20 ± 0.04 ^a^	35.09 ± 0.17 ^a^	35.15 ± 0.04 ^a^
S	19.96 ± 0.02 ^a^	20.06 ± 0.05 ^a^	19.58 ± 0.06 ^b^	S/T (%)	19.45 ± 0.17 ^a^	19.68 ± 0.03 ^a^	19.48 ± 0.05 ^a^
D	77.33 ± 0.02 ^a^	77.08 ± 0.08 ^a^	75.77 ± 0.07 ^b^	D/T (%)	75.33 ± 0.29 ^b^	75.59 ± 0.06 ^a^	75.38 ± 0.17 ^b^
TFT	969.44 ± 0.61 ^a^	963.13 ±0.44 ^b^	946.90 ± 0.67 ^c^	TST	45.19 ± 0.35 ^a^	45.95 ± 0.05 ^a^	44.45 ± 0.32 ^b^
TBT	472.04 ±0.33 ^b^	475.80 ± 0.73 ^a^	460.31 ± 0.59 ^c^	HAA	53.50 ± 0.46 ^a^	53.17 ± 0.21 ^a^	52.50 ± 0.51 ^b^
PER1	6.96 ± 0.01 ^a^	6.89 ± 0.03 ^ab^	6.76 ± 0.12 ^bc^	PER2	7.02 ± 0.03 ^a^	6.96 ± 0.04 ^a^	6.83 ± 0.01 ^b^
PER3	8.11 ± 0.01 ^a^	8.18 ± 0.06 ^a^	7.83 ± 0.08 ^b^	——	——	——	——

Note: Total amino acids, TAA; total essential amino acids, EAA; total non-essential amino acids, NEAA; total hydrophobic amino acids, HAA; total flavourful amino acids, F; total bitter amino acids, B; total sweetened amino acids, S; total medicinal amino acids, D; total flavourful amino acid TAV value, TFT; total bitter amino acid TAV value, TBT; total sweetened amino acid TAV value, TST; fresh amino acids are denoted as “*”; bitter amino acids are denoted as “**”; sweet amino acids are denoted as “^#^”; medicinal amino acids are denoted as “^##^”; taste thresholds for Glu, Asp, Arg, His, Met, Ile, Leu, Phe, Trp, Val, Ala, Gly, Ser, Thr were 30, 100, 10, 20, 30, 90, 380, 150, 90, 150, 60, 110, 150, 260 (mg/100 g). Taste activity value (TAV) was used to evaluate the flavour of coix seed, which is the ratio of the amount of each flavour-presenting amino acid in a sample to its corresponding flavour threshold. When TAV > 1, the flavouring substance is considered to have a significant effect on the flavouring of the sample, while when TAV < 1, the substance has no significant flavouring effect. Different letters (a–c) indicate significant difference between the mean values of different samples (*p* < 0.05).

**Table 4:** Secondary structure composition of interactions between proteins and polyphenols of CSP.

Sample	FTIR				Raman			
	α-Helix	β-Sheet	β-Turn	Random Coil	α-Helix	β-Sheet	β-Turn	Random Coil
Raw	20.87 ± 0.30 ^c^	48.50 ± 1.12 ^a^	19.73 ± 0.25 ^a^	10.91 ± 0.28 ^b^	23.13 ± 0.56 ^c^	48.73 ± 0.17 ^a^	17.40 ± 0.38 ^a^	10.74 ± 0.22 ^c^
Stir-frying	21.88 ± 0.13 ^bc^	49.78 ± 0.57 ^a^	14.18 ± 0.51 ^b^	14.15 ± 0.31 ^a^	24.30 ± 0.14 ^b^	44.36 ± 0.75 ^b^	16.60 ± 0.12 ^ab^	14.74 ± 0.13 ^b^
Bran stir-frying	22.01 ± 0.45 ^a^	49.94 ± 0.78 ^a^	13.48 ± 1.05 ^b^	14.56 ± 0.12 ^a^	25.91 ± 0.39 ^a^	45.14 ± 1.01 ^b^	12.23 ± 0.47 ^c^	16.73 ± 0.09 ^a^

Note: Different letters (a–c) indicate significant difference between the mean values of different samples (*p* < 0.05).

## Data Availability

The data used to support the findings of this study can be made available by the corresponding author upon request.

## Appendix A

**Table A1:** Peptide and activity prediction of coix seed prolamins after digestion.

Protein Name	Sequence (Results of Enzyme Action)	Molecular Weight	Length	Protein Molecular Structures	DH (%)
α-coixin	MATK - IF - AL - L - VL - L - AL - SASATTAVIIPQCSL - APTAAIIQR - F - L - PPVSAIGF - EHPAVQAYR - L - QQAL - AATIL - QQPL - AQL - QQR - SSAHL - TIQTIAAQQQQQQF - L - ASL - SQL - AAANPAAYL - QQQL - L - ASNPL - AVANAIEYQQQQQL - QQF - L - PAL - SQL - VVANPAAYL - QSQL - F - PSNPL - VVTNSAAYL - QQQQL - QQIL - PAL - SQL - AVANPNSYL - QQQQL - L - PF - NQVAVANNAVYQQQYQL - L - QVNPL - VATF - L - QQQQR - QL - L - PF - NQMSL - MNPAL - SWQQPIVGGVGF	29.0 KDa	266		21.5094
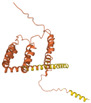
α-coixin 7	MAAK - IF - AL - L - AL - L - AL - STNATATF - F - PQCSPVTTTIPQYL - PSVVATR - F - EYPVSQSHR - L - QEAL - AASIL - QSL - VVIL - R - QPYSL - L - QQPSL - ANL - F - VQSIAAQQL - QQQL - L - PAINQVDAANL - AAYL - QQQQF - L - PYNQL - AR - VNPAAYL - QAQL - L - PF - NQQL - AVASPIASL - QQQL - L - PF - YPQAL - AQTVAF - L - QQQQQQQQQL - L - PF - YPQAMANIAAF - L - QQQQQL - L - PF - YPNDVANIVAL - L - QQQQL - L - PF - SQL - AL - R - NPTAL - L - QTQQL - L - PF - YSQVVANIAAF - L - QQQQL - L - PF - SQL - VL - R - NPATL - L - QQAIIGGAIF	32.3 KDa	291		26.2069
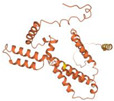
α-coixin 8	MAPQIF - SL - L - ML - L - VL - CASAATATIIPQCSQR - PL - L - PPF - L - PSVTTSICENQIL - QPYR - L - QEAIAESIL - R - SSPL - F - AQQPL - AL - L - QQL - PL - VNF - L - AQNIR - AQQL - QQL - VL - PAISQVAMANL - AAYSQQQQF - L - PF - NQQANL - VAYSQQQQF - L - PF - NQL - ANL - IAYSQQQQF - L - SF - NQL - VAVNPAAYL - QQQQL - L - L - PF - SQL - AAASPAAF - L - PQQQL - L - SF - YPQAL - ANAASIQL - QQL - L - PL - IQL - AL - R - NPAASCQQPIVGAAL - F	26.6 KDa	242		26.1411
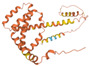
β-coixin	MK - MVIVL - AVCL - AL - SAAASASAL - QMPF - L - AGL - QGL - YGAGDL - MTTTMMMGAGGGL - YPCAEYL - R - QPQCSCSPVAVPYYAQQR - EQMTMCQQMR - MMGMQSR - CGAMCGVAQQSVAQQL - QMMMQL - QGTAAAASSGL - L - YEPAL - MQQL - L - PAVAAQAAL - TNPMAMAVAQAAQNMPAAMCGL - YQQL - PSYCGTTPSCAVSAAIPPYY	20.2 KDa	194		13.4715
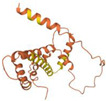
α-coixin 27 kDa	F - AL - L - VL - L - AL - SASATTAVIIPQCSL - APTAAIIPR - F - L - PHVSAIGF - EHPAVQAYR - L - QQAL - AASIL - QQPL - AQL - QQQSSAHL - TIQTIAAQQQQF - L - PSL - SQQAAVNPAAYL - QQQL - L - ASNPL - AVPNAIAYQQQQQL - QQF - L - PAL - SQL - AVANPAAYL - QSQL - F - PCNPL - VANAAAYL - QQQQL - QQIL - PAL - SQL - AVANPNSYL - QQQQL - L - PF - NQVAVANNAVYEQQHQL - L - QVNPL - AAAF - L - QQQQR - QL - L - PF - NQMSL - MNPAL - SWQQPIVGGVGF	27.9 KDa	258		21.0117
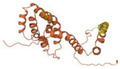
γ-coixin	MK - EVL - VVL - AF - MAL - VASAACTQTCACSCGQQQSHEQQHHHPPQHHPQK - QQHQPPPPQK - QHHQQQVHMEQK - QHHQQQEVVHQPQEVHVK - QQPQHQEQHR - CEGQQQQHQQSQGHGQQQGQSHEQQQCHGQETPQQHQQQCQGYMQHQPPQYQQCQQGHEQPQQQQCYCQENPL - QQPK - QCQK - GCEQQQTTK - CSYSYYSGNSNL - K - NCHEF - L - R - QQCNPL - VMPF - L - QSR - L - VQPSNCQVL - R - QQCCHEL - R - QIEPQYL - HQMIYSMVQSTIQQQQQQQQQQQQSDEL - WGF - QL - PIQSEVAIL - TAAQYL - PSMCGL - YHSCYPNNPYSSYDATGGVR - N	37.6 KDa	322		11.2150
